# Willing to wait: Anorexia nervosa symptomatology is associated with higher future orientation and reduced intertemporal discounting

**DOI:** 10.1038/s41598-024-80597-7

**Published:** 2025-02-06

**Authors:** Isabel Schuman, Jingyi Wang, Ian C. Ballard, Regina C. Lapate

**Affiliations:** 1 Department of Psychological & Brain Sciences, University of California, Santa Barbara, Santa Barbara, USA; 2 Department of Psychology, University of California, Riverside, Riverside, USA

**Keywords:** Psychology, Human behaviour, Psychiatric disorders

## Abstract

Anorexia nervosa is a severe eating disorder characterized by food restriction in service of a future goal: thinness and weight loss. Prior work suggests altered intertemporal decision-making in this disorder, with more farsighted decisions—i.e. reduced delay discounting—in patients with acute anorexia nervosa. Future-oriented cognition, such as frequent prospective future thinking in daily life, promotes farsighted decision making. However, whether temporal orientation is altered in anorexia nervosa, potentially contributing to reduced delay discounting in this population, remains unclear. We measured delay discounting behavior, anorexia nervosa symptomatology, and temporal orientation in a large sample of never-diagnosed individuals. We found that higher anorexia nervosa symptomatology was associated with reduced delay discounting. Anorexia nervosa symptoms were also correlated with increased future-oriented cognition. Moreover, future-oriented cognition mediated the difference in delay-discounting behavior between high and low anorexia nervosa symptom groups. These results were unrelated to subjective time perception and independent of mood and anxiety symptomatology. Collectively, these findings suggest that future-oriented cognition may be a cognitive mechanism underlying altered intertemporal decision making in anorexia nervosa.

**Supplementary Information:**

The online version contains supplementary material available at 10.1038/s41598-024-80597-7.

## Introduction

### Overview

The ability to guide behavior according to long-term goals is often adaptive—it is linked to higher academic performance, professional success, and favorable health outcomes^1,2^. For instance, the propensity to prefer smaller rewards now as opposed to larger rewards in the future—termed delay discounting—is associated with increased incidence of substance abuse, gambling, bipolar disorders, and depression (for reviews, see:^2–4^). A notable exception to this seemingly advantageous decision-making profile is seen in *Anorexia Nervosa*, a severe eating disorder characterized by persistent restrictions in calorie intake in service of a subjectively preferred future goal: thinness and weight loss^5^, which culminates in extremely low body weight, long-lasting impairments physical and mental health, and one of the highest mortality rate amongst psychiatric conditions^6,7^. Efficacious future-oriented control of behavior, combined with altered reward processing, may underlie the unique capacity of patients with anorexia nervosa to self-starve despite the inherently rewarding nature of food^8–12^. Accordingly, delay discounting is often reduced in patients with acute anorexia nervosa compared to healthy controls^13–17^, which contrasts with findings in nearly all other psychiatric disorders^2,4^.

However, the cognitive mechanisms underlying the association between anorexia nervosa and reduced delay discounting remain to be fully determined. How may information be processed differently in patients with anorexia nervosa, such that future rewards are not as sharply discounted as they are for most individuals? A growing body of work on intertemporal discounting suggests that a future-oriented cognitive style—i.e., the tendency to spontaneously consider the future when making decisions—may be linked to less steep discounting of future rewards by facilitating the representation of future decision outcomes via episodic prospection or future “time travel”^3,18–22^, an idea that has not been tested in individuals with anorexia nervosa symptomatology.

### Anorexia nervosa and intertemporal discounting

Delay discounting paradigms model choice preferences in one’s subjective evaluation of present and future rewards^2,3,23^. Concretely, volunteers are typically asked to choose between two hypothetical monetary amounts (e.g., $20 now or $30 in one week), which vary in value and temporal availability. By modeling choice behavior across hundreds of trials, a reliable discounting rate—reflecting the degree to which an individual discounts the value of a monetary reward as the delay to reward receipt increases —is obtained per individual.

Delay discounting rates are predictive of impatience and impulsivity in domains that involve making trade-offs between present and future rewards—such as academic and professional performance, substance use, exercise, and health behaviors^24–30^. While healthy individuals typically prefer smaller-but-sooner rewards—and discount larger-but-later rewards according to a hyperbolic function^31^—individuals with acute anorexia nervosa often show *lower* discounting rates compared to healthy controls and individuals diagnosed with other disorders^13–17,32^ (for a review, see^33^). For instance, Steinglass (2017) found that patients with anorexia nervosa discounted monetary rewards less steeply than healthy controls, unlike patients with social anxiety or obsessive compulsive disorders^14^.

While delay discounting is a promising measure for characterizing decision making abnormalities across several distinct psychiatric conditions^2,4^, upon weight restoration or recovery from acute anorexia nervosa, delay discounting rates have been found to return to those of healthy controls^15,17,34,35^. Thus, it is possible that reduced delay discounting in patients with anorexia nervosa may result from cognitive alterations associated with acute starvation—or, alternatively, that in-patient eating disorder interventions may themselves alter (and correct) intertemporal decision-making behaviors. Therefore, examining delay discounting in individuals showing varying levels of eating disorder symptomatology—including in never-diagnosed samples—is required to begin to elucidate whether intertemporal decision making may be a vulnerability factor for anorexia nervosa.

### Intertemporal discounting mechanisms: temporal processing alterations in anorexia nervosa?

Prior empirical and theoretical work in healthy samples highlights three component processes that jointly contribute to temporal choice patterns: reward valuation, cognitive control, and prospective future thinking^2,3,36–39^. Prospection is the process of imagining possible future episodes and outcomes^40^ and is thought to play a critical role in future reward evaluation and facilitate cognitive control of behavior toward long-term goals^41^. The propensity to mentally time travel into future episodes when making decisions (also called episodic future thinking) permits distal outcomes to be simulated and experienced more vividly and concretely, and “engenders a greater consideration of the future consequences of the choices one makes in the present”^3^. Indeed, a growing body of work underscores that the way time is represented and incorporated during decision making—including the tendency to consider future consequences during everyday life decisions, the vividness of future imagery, and the accuracy of time estimation—shapes temporal discounting behavior. For instance, context manipulations that promote envisioning temporally distal rewards more concretely reduce delay discounting, presumably by lending them heavier weight when considering intertemporal trade-offs^42–45^. Consistently, individuals who show a future-oriented thinking style—characterized by the propensity to consider future consequences (*versus* considerations about the present or the past) typically show reduced delay discounting (as measured by the Zimbardo Time Perspective Inventory (ZTPI)^21,46^ and the Consideration of Future Consequences (CFC)^18–20,22,47^). A prior study examining a clinical sample found that individuals with anorexia nervosa display increased future-oriented cognition as measured by the ZTPI^48^. However, whether future-oriented cognition is associated with higher anorexia nervosa symptomatology— and whether that may account for altered delay discounting behavior in symptomatic individuals—remains unknown.

During prospective thinking, the accuracy of temporal (e.g., delay) representations has itself been suggested to modulate intertemporal choice behavior, such that the overestimation of time may result in the devaluing of future (vs. immediate) rewards^39^. Accordingly, overestimation of future durations is associated with steeper delay discounting rates and increased preference for immediate rewards^38,49^. Relatedly, impulsive individuals typically over-estimate the duration of temporal intervals^50–52^ (reviewed in^39^). Although few empirical studies have explored temporal processing alterations in eating disorders, a recent study found that adolescents with anorexia nervosa under-estimated temporal durations compared to healthy controls^53^, raising the possibility that subjective time perception may be associated with anorexia nervosa symptoms.

### The current study

Collectively, the above-reviewed findings in clinical samples raise the possibility that inter-temporal discounting may be altered in individuals with higher anorexia nervosa symptomatology, putatively due to alterations in future-oriented cognition—a set of hypotheses we tested in this pre-registered study (https://osf.io/h46x2). First, we aimed to extend the prior literature indicating low intertemporal discounting in individuals with acute anorexia^13–17^ to a not-previously diagnosed community sample with high versus low levels of anorexia nervosa symptomatology. Building on prior findings, we hypothesized that individuals with higher anorexia nervosa symptoms would show reduced intertemporal discounting [log(*k*)]. We additionally estimated intertemporal discounting using a newer computational model (Ebert-Prelec^37,54^) that parsed intertemporal discounting into impatience (‘*a*’) and time sensitivity (‘*b*’) parameters to examine whether they provided additional insights into specific decision-making processes that may be altered in anorexia nervosa.

Second, we sought to investigate whether future-oriented thinking is associated with anorexia nervosa symptoms, potentially accounting for reduced intertemporal discounting in high-symptom individuals. To that end, we obtained a latent score indexing individual differences in future-oriented cognition using two well-validated self-reported questionnaires (the Zimbardo Time Perspective Inventory (ZPTI) and the Consideration of Future Consequences (CFC)) and a temporal orientation task (Preoccupation with Future Events; PFE). We hypothesized that individuals with higher anorexia nervosa symptoms would show increased future oriented cognition.

**Fig. 1:**
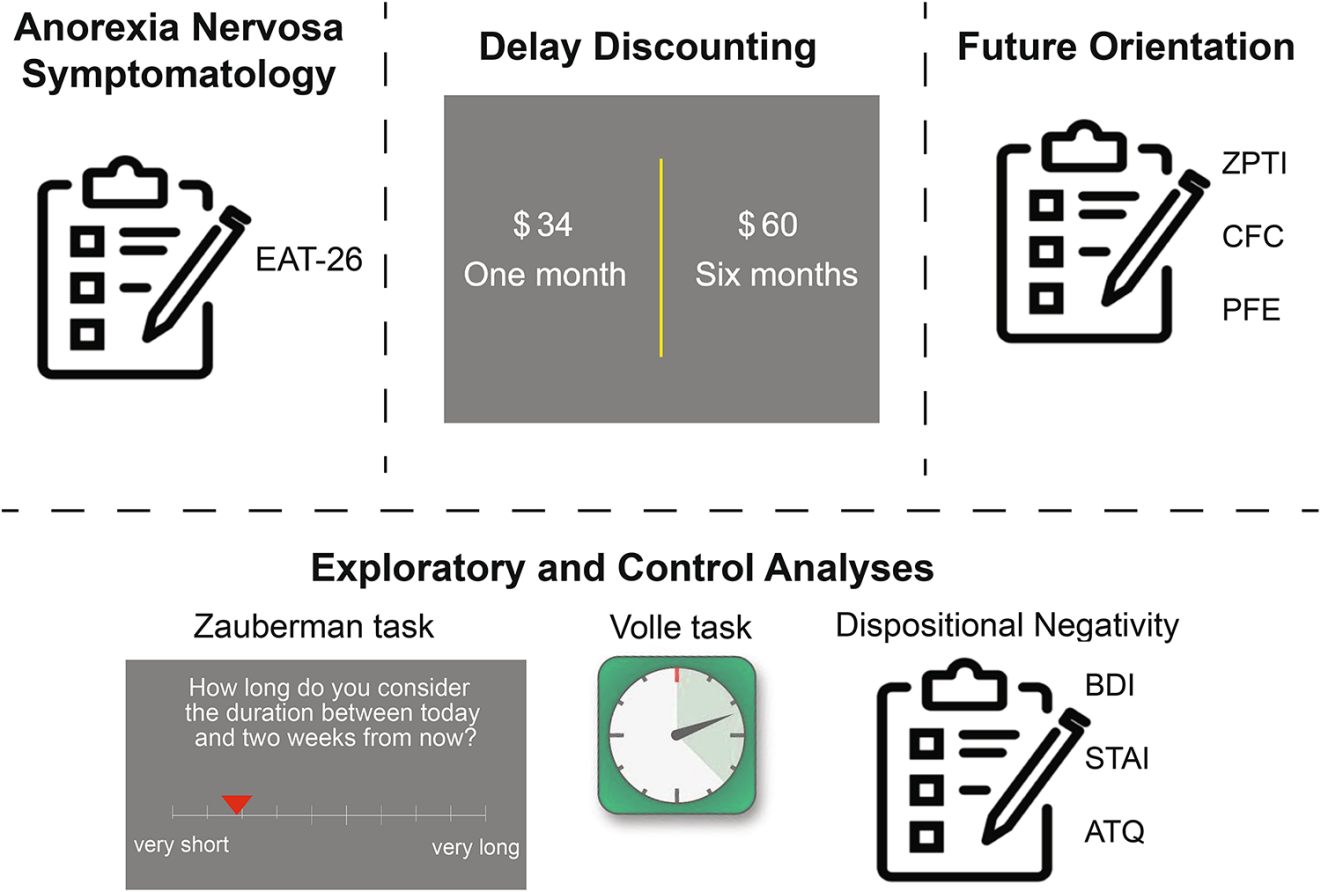
Overview of experimental procedures. The Eating Attitudes Test (EAT-26) questionnaire was used as a metric of participants’ anorexia nervosa symptomatology. To index intertemporal behavioral choices, participants completed a delay discounting task. Future-oriented cognition was measured using a latent factor derived from the data of 2 questionnaires (ZTPI & CFC) and one task (PFE). Pre-registered exploratory and control analyses included subjective time measures (the Volle and Zauberman tasks) as well as dispositional negativity, a factor score derived from mood and anxiety questionnaires (BDI, ATQ, and STAI-T).

In light of prior evidence suggesting that the way time is subjectively experienced modulates inter-temporal choice^39^, we included tasks assessing subjective temporal processing accuracy in our study, which were examined in pre-registered exploratory analyses. Finally, because anxiety and depression are often comorbid with anorexia nervosa, and have been associated with intertemporal choice^14^, participants completed well-validated mood questionnaires (Beck Depression Inventory (BDI)^55^, Spielberger State-Trait Anxiety Inventory (Trait scale: STAI-T)^56^, and the Adult Temperament Questionnaire: Negative Affect subscale^57^) to disentangle the potential contributions of mood and anxiety from the hypothesized associations between anorexia nervosa symptomatology, future-oriented cognition, and intertemporal choice (Fig. 1).

## Methods

### Participants

Due to COVID-19 related University closures when data collection for this study began (Spring 2021), data were collected online using Pavlovia (task data; https://pavlovia.org) and Qualtrics (questionnaire data; https://www.qualtrics.com). A preregistered sample of *n* = 152 individuals (Age range = 18–51; *M* = 27.33, *SD* = 9.68; *n* = 109 females, 40 males, 3 non-binary; *n* = 1 chose not provide demographic information) completed the experiment (See sample size justification in the preregistration: https://osf.io/h46x2). Fifty-seven of those individuals fell in the anorexia nervosa high-symptom group (Age *M* = 30.49, *SD* = 10.01; *n* = 43 female, 11 male, 3 non-binary) and *n* = 95 in the low-symptom group (Age *M* = 25.41, *SD* = 8.99; *n* = 66 female, 29 male). To maximize data quality and verify task compliance in this online study, data cleaning procedures were performed prior to each analysis according to pre-registered criteria (for details, see Data quality criteria in Supplementary Methods; demographic information for participants included in each analysis can be found in Supplementary Table 1) Participants were recruited from UC Santa Barbara using SONA (*n* = 62) and from the broader community using Prolific (*n* = 90). Eligible participants were fluent English speakers, with normal or corrected-to-normal vision, and at least 18 years of age. Written informed consent was obtained from all participants. All procedures were approved by the Human Subjects Review Committee at the University of California, Santa Barbara. Participants were compensated for their participation with course credit or payment. All methods were carried out in accordance with relevant guidelines and regulations.

### Materials & procedures

#### Eating disorder symptoms & group assignment

We assessed eating disorder symptoms using the *Eating Attitudes Test (EAT-26)*, a well-validated 26-item questionnaire^58,59^. For each item, participants indicate how often they experience each thought or action, ranging on a 5-point Likert scale from “Never” to “Always”. For items 1–25, “Never, Rarely, Sometimes” are coded as 0, “Often” is coded as 1, “Usually” is coded as 2, and “Always” is coded as 3. For item 26, “Always, Usually, Often” are coded as 0, “Sometimes” is coded as 1, “Rarely” is coded as 2, and “Never” is coded as 3. Scores above 20 indicate that an individual is “high risk” and scores below 20 indicate “low risk”^58^. Based on this criterion, participants were assigned to either the high or low anorexia nervosa symptomatology group (henceforth “high symptom” and “low symptom” groups) (M_High−symptom_ = 28.179, SD_High−symptom_ = 8.894 vs. M_Low−symptom_ = 5.745, SD_Low−symptom_ = 4.441; see Supplementary Table 4 for mean and standard deviation of key demographic and experimental variables examined in this study as a function of anorexia nervosa symptom group (high vs. low)).

Of note, the EAT-26 was originally validated on a sample of patients with anorexia nervosa^58^. While this questionnaire has excellent sensitivity and specificity for DSM-IV eating disorders diagnoses^59^, it was not designed to discriminate between anorexia and bulimia nervosa. Therefore, while we refer to eating disorders symptoms as measured by the EAT-26 in this study as “anorexia nervosa symptomatology” (high vs. low) due to the historical origin of the questionnaire, in addition to the growing recognition that anorexia and bulimia nervosa may exist on a continuum^60–62^, the current study cannot fully discriminate between anorexia nervosa (e.g., restrictive vs. purging) subtypes, nor between anorexia and bulimia nervosa (but see “Further ascertaining the sensitivity of delay discounting metrics to anorexia nervosa symptoms” for a re-analysis of the data after excluding bulimia-nervosa related EAT-26 items prior to symptom-based group assignment).

#### Disposable income

Participants self-reported their monthly disposable incomes using the following scale: 1: 0-$500, 2: $500-$1000, 3: $1000-$1500, 4: $1500-$2000, 5: $2000-$2500, 6: $2500-$3000, 7: $3000+.

#### Future orientation

The following questionnaires were used to derive the latent factor Future Orientation, which was used for subsequent analysis: Consideration of Future Consequences Scale^47^, the Zimbardo Time Perspective Inventory^46^ and Preoccupation with Future Events^63^. These questionnaires are described below. Please see Supplementary Table 5 for the validity and reliability metrics of the questionnaires adopted in this study.

##### Consideration of Future Consequences

The extent to which participants self-report taking future consequences into consideration during everyday-life decision making was measured using the Consideration of Future Consequences Scale (CFC)^47^. The CFC is composed of 12 statements and participants are asked to indicate how characteristic of them they find each statement to be, ranging from “extremely uncharacteristic” to “extremely characteristic”. For items 1, 2, 6, 7, and 8, “extremely uncharacteristic” is coded as 1, “somewhat uncharacteristic” is coded as 2, “uncertain” is coded as 3, “somewhat characteristic” is coded as 4 and “extremely characteristic” is coded as 5. For items 3–5 and 9–12 the scores are reversed. Scores on each item were summed to produce a total score ranging from 12 to 60, with higher scores reflecting greater consideration for future consequences.

##### The Zimbardo Time Perspective Inventory

Participants’ temporal orientation—i.e., the extent to which they take future goals (versus, for instance, enjoyment of the present-moment) into account when making decisions—was measured using the Zimbardo Time Perspective Inventory (ZTPI)^46^. The ZTPI is a 56-item questionnaire composed of 5 individual subscales representing different temporal orientation factors, including past-negative, past-positive, present-hedonistic, present-fatalistic, and future. Participants were asked to indicate how characteristic of themselves each statement was, on a five-point scale ranging from 1 (extremely uncharacteristic) to 5 (extremely characteristic). A higher score represents a greater orientation towards that factor. Average scores were obtained for the future-orientation subscale.

##### Preoccupation with Future Events

Participants’ preoccupation with future events was assessed using a procedure following Klineberg (1968)^63^. Participants were asked to write down ten things that they thought about in the prior two weeks. Next, participants examined each thought they wrote down, and indicated whether that thought primarily pertained to the past, present, or future when they were thinking about it. Preoccupation with future events was determined for each participant by calculating a ratio of future thoughts relative to total thoughts listed.

#### Dispositional negativity

Given the comorbidity of anorexia and mood and anxiety symptoms, participants completed three well validated trait mood and anxiety questionnaires, which were used to derive a Dispositional Negativity latent factor: the Beck Depression Inventory (BDI)^55^, the Spielberger State-Trait Anxiety Inventory (Trait scale: STAI-T)^56^, and the negative affect (NA) subscale from the short-form of the Adult Temperament Questionnaire (ATQ)^57^.

#### Delay discounting task

Intertemporal discounting was assessed by asking participants to make a series of hypothetical binary choices between two monetary rewards that differed in their amount and receipt time. For each trial, individuals chose between a reward that was lower in value but would be received earlier in time (“smaller sooner”), versus a reward that was greater in value but would be delivered after a delay (“larger later”). The temporal discounting task was modeled after McClure et al. (2004) and Decker et al. (2015)^15,23^. The smaller-sooner rewards ranged from $15-$85. The relative difference in reward magnitudes between smaller-sooner and larger-later rewards (r_larger−later_/r_smaller−sooner_ -1) ranged from 2 to 100%. Thus, larger-later rewards varied between $16-$170. This experimental choice set follows prior work as well as internal piloting, and was carefully selected to produce enough response variability required to estimate individual discount rates. Delay times until receipt of the smaller-sooner reward were either today, 2 weeks, or 1 month, which were randomly selected. Delay times to the larger-later reward ranged from 1 week to 6 months in the future and were always greater than the delay times to the smaller-sooner reward (see Supplementary Table 3). The screen position of smaller-sooner and larger-later reward options (i.e., to the left vs. right side of the screen) was counterbalanced across trials. Choice trials were self-paced. Participants selected their responses using a button press (“left” vs. “right” key). Each choice was followed by a 1.5 s inter-trial interval. The experiment contained *n* = 144 task trials (plus an additional *n* = 2 catch trials for participants run using the SONA system, and *n* = 10 catch trials for participants run using Prolific) and took approximately 30 minutes to complete. Participants were offered three breaks during the task. Participants were instructed that there were no right or wrong answers, and were asked to indicate which choice they would genuinely prefer to receive. Note that rewards used in this study were hypothetical rewards, which are known to produce behavioral choices that are highly correlated with behavioral choices for real rewards^64,65^.

#### Temporal accuracy tasks

We examined participants’ temporal processing accuracy using two behavioral tasks: the Volle Time Estimation Task^66^ and the Zauberman Future Time Estimation Task^49^. Because these tasks had not been previously used in a study of an eating disorder population to the best of our knowledge, these analyses were pre-registered as exploratory.

##### Volle time estimation task

In this silent counting task (adapted from^66^), participants were asked to estimate the passage of time until a specific target time interval (ranging from 20 to 50 s) was reached. Sequential numbers (ranging from 1 to 10) were flashed on the computer screen (0.1 s/each) at a given frequency—every 1s or every 2s—with the target time interval displayed on the lower right corner of the screen. Flashing numbers disappeared after 10 digits. Participants were asked to continue to silently count according to the flashing numbers’ frequency until they reached the target time interval. For the first 4 trials, flashing numbers were displayed every 1s (1 Hz), with the following target time intervals: 20, 30, 40, and 50. For the last 4 trials, numbers flashed every 2s (0.5 Hz) with the following target time intervals: 15, 20, 25, and 30. Time estimation accuracy was obtained by calculating the difference between participants’ time duration estimates relative to the actual (objective) target interval time for each trial.

##### Zauberman future time estimation task

In this task adapted from Zauberman et al. (2009)^49^, participants were asked to consider various time intervals from the present day—ranging from 1 week to 10 years into the future—and to indicate how long they perceived each time interval to be. To do so, they placed a marker using a mouse on a slider that was anchored on the labels “very short” (left) and “very long” (right). The time intervals to be estimated were presented sequentially, with the shortest one (i.e. 1 week from today) asked on the first trial. To facilitate participants’ usage of the scale, they were told at the beginning of the task that the largest time interval they would be asked about would be 10 years from the present date. We calculated the growth ratio (i.e., subjective/objective passage of time) by assessing individual’s prospective time estimation for each trial. Using “1 week” as the anchor, we computed objective growth as relative time differences between each trial vs. 1 week [i.e., (Time_trial_ – Time_anchor_)/Time_anchor_]. Accordingly, subjective growth was computed as the relative slider response differences between each trial and 1 week [i.e., (Slider response_trial_ – Slider response_anchor_)/Slider response_anchor_]. Growth ratio was computed by dividing subjective growth by objective growth, such that growth ratios closer to 1 indicate more accurate prospective time estimates.

#### Data processing and analysis

##### Future orientation: latent factor analysis

A principal component analysis (PCA) was run to derive a future-orientation latent factor from the above-described future orientation questionnaires and task (ZPTI, CFC and PFE). We used singular value decomposition-based PCA with varimax rotation (principal function; Psych R package)^67^. The first component of the three-questionnaire solution accounted for 54.6% of the variance of the questionnaire data, with loadings as follows: λ_cfc_ = 0.856; λ_ztpi_future_ = 0.864; λ_PFE_future_ = 0.398. Because λ_PFE_future_ had a lower-than-expected loading, we additionally ran a second PCA using the CFC and ZTPI scales only. We found that these two questionnaires explained 78.5% of the variance of the first latent factor, with loadings as follows: λ_cfc_ = 0.886; λ_ztpi_future_ = 0.886. Given the improved latent factor solution, we examined whether our results remained consistent when considering the two-questionnaire factor scores; all significant results reported below remained consistent when using the two (vs. pre-registered three-questionnaire-based) factor score (See Supplementary Tables 6 and 7 for details). Therefore, we report the results with our pre-registered three-questionnaire latent score analysis. Individual differences in factor loadings were examined in relation to temporal discounting parameters [log(*k*)] and anorexia nervosa symptoms (EAT-26 scores), as detailed below.

##### Dispositional negativity: latent factor analysis

We ran a factor analysis (PCA) using scores obtained in the BDI, STAI-T and ATQ-NA to obtain a latent factor indexing negative mood and anxiety, which we term dispositional negativity. The three questionnaires collectively explained 77.7% of the variance of the dispositional negativity factor, and all had loadings above 0.8 (λ_BDI_ = 0.871; λ_STAI-T_ = 0.935; λ_NA_ = 0.836).

##### Delay discounting task

As the primary analysis of the delay discounting task, we fit participants’ choice data using a hierarchical Bayesian model implemented in Vincent’s (2016) delay discounting toolbox 1.7.1 (https://drbenvincent.github.io/delay-discounting-analysis/)^68,69^. This approach has the benefit of increasing the reliability of parameter estimates by estimating group parameter distributions to constrain the estimates of individual discounting parameters. Participants’ subjective values (*SV*) of each of the two options were modeled using the hyperbolic discount function. This function discounts the value of delayed rewards according to the equation:

$$\:SV=\frac{A}{1+kt}$$

where A is the reward amount, *t* is the time to the reward and *k* is the discount rate. The toolbox generates a natural log-transformed discount rate (log(*k*)) for each participant, which normalizes the distribution of discount rates. Decisions were modeled using the default function in the package, which models decisions as a noisy function of the difference in subjective values between the two options:

$$\:p\left(Choose\:LL\right)=\epsilon\:+\left(1-2*\epsilon\:\right)\varvec{\phi\:}\left(\frac{{SV}_{LL}-{SV}_{SS}}{\alpha\:}\right)$$

where $\:\varvec{\phi\:}$ is the cumulative normal function, *ε* is a noise parameter governing the likelihood of random responding (higher *ε* indicates more random choices), and α is a noise parameter governing the acuity of the value comparison (as higher α values reflect reduced sensitivity to the subjective value difference between the options).

We fit low and high-symptom groups separately, following the traditional analysis pathway for hierarchical temporal discounting analysis^69^. Because hierarchical parameter fitting assumes that all participants’ data arise from the same group distribution, the alternative approach of fitting all participants as one group could have spuriously influenced the differences between the low and high symptom groups^70,71^. Notably, the approach we used of estimating group-level parameters separately for different subject populations is also used by hierarchical cognitive modeling tools in other domains^70,72,73^. Through piloting, we designed our choice set to sample the range of discount rates observed in typical subject samples (with unknown anorexia symptomatology). We incorporated the prior expectation that our choice set would evenly sample discount rates into the model by setting the prior on the group log(*k*) to a value that would predict an approximately equal number of larger-later and smaller-sooner preferences in our choice set. We used the package default for the variance of the prior, resulting in a *N*(-5.3, 2.5) prior on log(*k*). Using Markov Chain Monte Carlo, we ran four independent sampling chains for 25,000 samples each and discarded the first 1000 samples of each chain as burn-in, resulting in 96,000 samples from the posterior distribution. To assess whether the sampling procedure converged to the posterior distribution, we computed the $\:\widehat{R}$ statistic, a measure of whether the four chains converged to the same distribution, for all parameters. No participant’s parameter values had $\:\widehat{R}$> 1.01, indicating chain convergence. The fitted model explains participant behavior well, correctly classifying 86.8% of choices. The mean and standard deviation of the key parameters of the model are reported in Supplementary Table 4.

Detailed Methods and Results for the Ebert-Prelec model are detailed in Supplemental Material (Supplementary Methods and Supplementary Results).

##### Primary analyses

Differences in discount rates [log(*k*)] and future orientation by anorexia nervosa symptom group (low, high) were examined using independent *t*-tests as the primary analysis given the established clinical significance of the EAT-26 cutoff value adopted here and elsewhere^58,59^. In alignment with the RDoc framework, we additionally examined linear associations between those variables using a continuous approach as additional analysis using Pearson’s correlation coefficients (see Supplementary Results).

Following our pre-registration, one-sided hypotheses regarding the association between anorexia nervosa symptom group, intertemporal discounting, and future orientation were tested using one-tailed *p*-values. Two-tailed *p*-values were used for analyses pre-registered as exploratory (temporal processing tasks) and/or for any analyses that were not originally pre-registered—such as dispositional negativity and the mediation analysis between anorexia nervosa symptom group, future orientation, and intertemporal discounting (see Supplementary Materials for a complete description of new analyses conducted beyond the pre-registered analysis plan).

##### Exploratory analyses

We tested whether time estimation accuracy (Volle task and Zimbardo task scores) varied by anorexia nervosa symptom group using independent sample *t*-tests. In addition, we examined whether anorexia symptoms (EAT-26 scores) correlated with temporal accuracy using Pearson’s correlation. As mentioned, all exploratory analyses were conducted using two-tailed *p* values.

Given the comorbidity of anorexia and anxiety disorders, we examined whether Dispositional Negativity varied by anorexia nervosa symptom group, and entered Dispositional Negativity as a covariate in a control analysis to test whether future-oriented cognition explained anorexia nervosa symptomatology after controlling for dispositional negativity scores.

Finally, because we observed the hypothesized group differences in intertemporal choice and future-oriented cognition as a function of anorexia nervosa symptomatology, and in light of prior work suggesting prospection and future-oriented cognition as an important mechanism underlying intertemporal choice, we conducted an exploratory mediation analysis to determine whether future-oriented cognition explained (i.e., statistically mediated) anorexia nervosa symptom group differences in intertemporal discounting. To do so, we used ordinary least squares regression via the PROCESS software version 4.3.1 in R (Hayes, 2022). Evidence for mediation was obtained using a bootstrap confidence interval (*n* = 10,000 samples) for the partial standardized indirect effect^74^.

##### Control analyses: gender, age, and income

We examined the possibly confounding influence of gender, age, and income in intertemporal discounting behavior, anorexia nervosa symptoms, and future-orientation (using log(*k*), EAT-26 scores, and future-orientation factor scores, respectively). We tested for potential gender differences using independent sample *t*-tests and age-related differences using Pearson’s correlation. We examined the impact of income differences on delay discounting, anorexia nervosa symptoms, and future-orientation using a one-way ANOVA.

##### Ascertaining the sensitivity of delay discounting to anorexia (vs. bulimia) nervosa

As mentioned above, the present study assessed anorexia symptomatology using the EAT-26, which was originally validated using a sample of patients with anorexia nervosa^58^, while including items related to bulimia nervosa (e.g. binge eating and purging behavior). Thus, we re-analyzed our data after excluding EAT-26 items measuring binge eating and purging behavior (items 4, 9, and 25). Participants were then assigned to the ‘high-symptom’ or ‘low-symptom’ group using the same EAT-26 cutoff described above (M_High−symptom_ = 26.732, SD_High−symptom_ = 8.064 vs. M_Low−symptom_ = 5.649, SD_Low−symptom_ = 4.362; see also Supplementary Table 4). Thus, this analysis allowed us to better ascertain whether and how anorexia nervosa symptomatology—now estimated after the exclusion of bulimia nervosa symptoms—was associated with intertemporal discounting and future orientation in this sample.

## Results

### Individuals with anorexia nervosa symptomatology show reduced intertemporal discounting

**Fig. 2:**
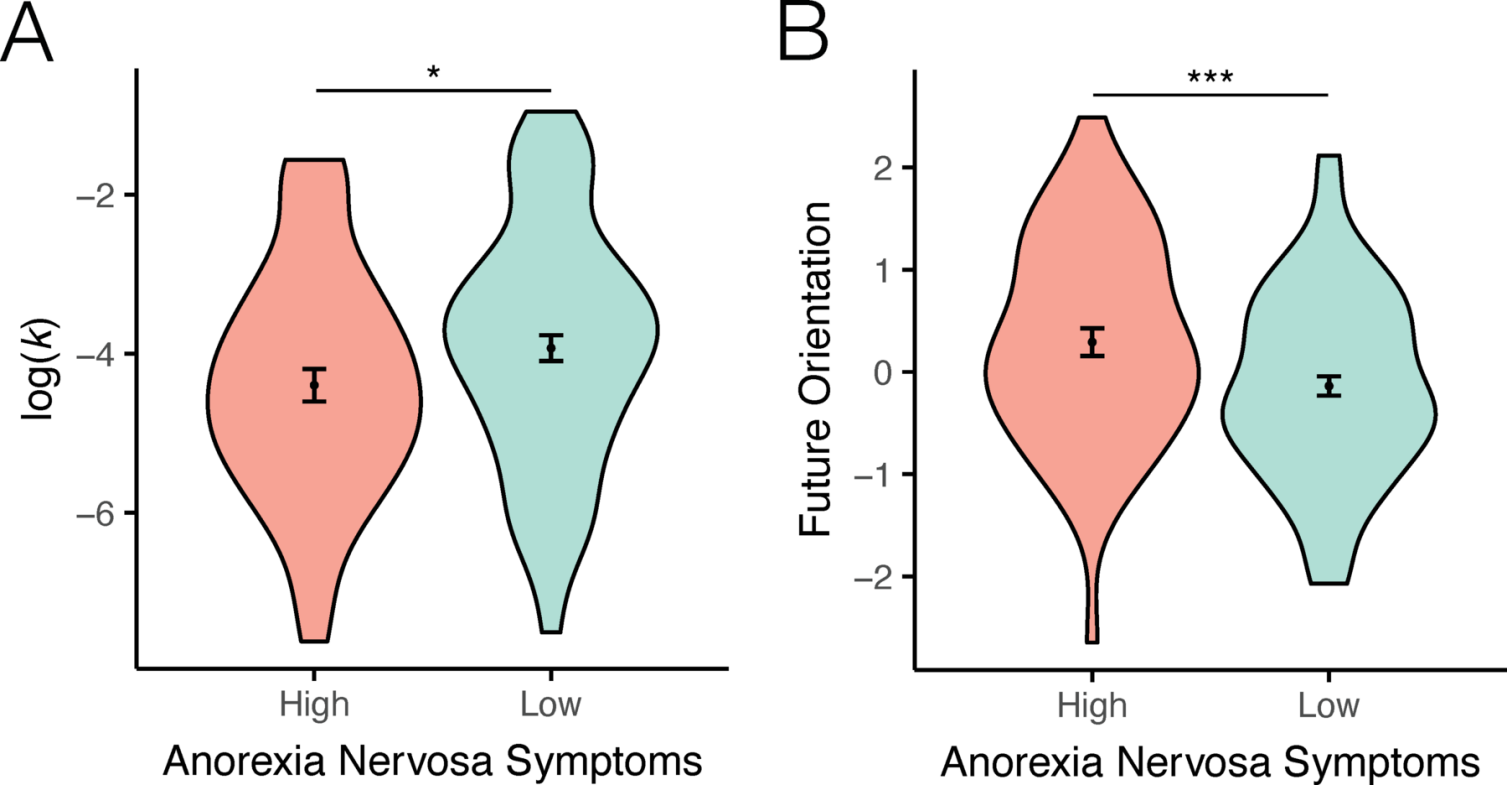
Intertemporal decision making (**A**) and temporal orientation (**B**) as a function of anorexia nervosa symptoms. (**A**) Individuals with higher anorexia nervosa symptoms show reduced delay discounting compared to a low-symptom group. (**B**) Anorexia nervosa symptomatology is associated with a future-oriented cognitive style. *p < 0.05, ***p < 0.005.

Individuals in the anorexia nervosa high-symptom group devalued future rewards less steeply than individuals in the low-symptom group, as indicated by significantly lower intertemporal discounting rates in high- vs. low-symptom groups (log(*k*) M_High−symptom_= −4.394, SD_High−symptom_ = 1.434 vs. M_Low−symptom_= −3.928, SD_Low−symptom_ = 1.514, *t*_(134)_= −1.758, *p* = 0.041, d_z_ = 0.314) (Fig. 2A).

### Individuals with anorexia nervosa symptomatology show increased future-oriented cognition

Next, we examined whether a putative cognitive mechanism underlying intertemporal discounting—the propensity to engage in prospective, future oriented thinking during everyday decisions—might be associated with anorexia nervosa symptomatology and explain group differences in intertemporal choice behavior. First, in agreement with prior work^18–21^, intertemporal discounting rates and future-orientation factor scores were negatively associated across the sample, such that higher future-oriented cognition predicted lower intertemporal discounting [log(*k*)] (*r*_(132)_=-0.26, Pearson’s *p* = 0.002). Critically, we found a significant group difference in future-oriented cognition by anorexia nervosa symptomatology, such that individuals in the high-symptom group had significantly higher future-orientation scores compared to the low-symptom group (M_High−symptom_= 0.292, SD_High−symptom_ = 1.007 vs. M_Low−symptom_= −0.138, SD_Low−symptom_ = 0.916, *t*_(147)_ = 2.659, *p* = 0.004, d_z_ = 0.451) (Fig. 2B).

### Future-oriented cognition mediates the association between anorexia nervosa symptomatology and intertemporal decision making

**Fig. 3:**
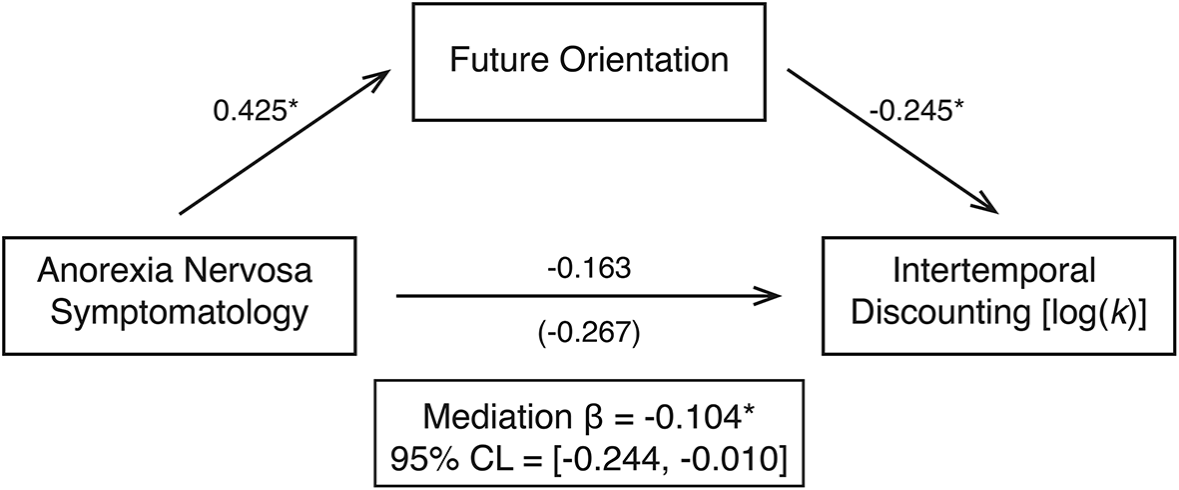
Future-oriented cognition significantly mediated the association between anorexia nervosa symptomatology and intertemporal discounting. Mediation paths with standardized regression coefficients are shown. The total effect relating anorexia nervosa symptom group and intertemporal discounting rate is shown in parenthesis. * *p* < 0.05.

In light of prior work suggesting that prospective, episodic future thinking may promote intertemporal behavioral choices, we examined whether the anorexia-nervosa related difference we observed in intertemporal discounting when comparing high- versus low-symptom groups was accounted for by group differences in future-oriented cognition. To that end, we conducted a mediation analysis (see Methods). As detailed in Fig. 3, individuals in the anorexia nervosa high-symptom group showed higher future-orientation scores than the low-symptom group (ß = 0.425, SE = 0.174, *t* = 2.389, *p* = 0.018). Moreover, a future-oriented cognitive style predicted reduced intertemporal discounting [log(*k*)] (ß = −0.245, SE = 0.131, *t* = −2.852, *p* = 0.005). Critically, a bootstrap analysis of the partial standardized indirect effect (ß= −0.104, SE = 0.061) indicated that future orientation mediated anorexia-symptom differences in intertemporal discounting [95% CI = (-0.244, -0.010)]. Accordingly, after controlling for future-orientation scores, we found that the association between anorexia symptom group and log(*k*) values was reduced (total effect: ß =−0.267, SE = 0.267, *t* = −1.482, *p* = 0.141, partial effect: ß = −0.163, SE = 0.266, *t* = −0.909, *p* = 0.365). In sum, these results suggest that a future-oriented cognitive style may underlie reduced delay discounting in individuals with high anorexia symptomatology.

### No reliable differences in subjective time perception by anorexia nervosa group

To test whether anorexia nervosa symptomatology modulated subjective time perception, we examined the performance of individuals showing high vs. low anorexia nervosa symptoms in two previously-validated temporal processing tasks: the Volle task and the Zauberman task (see Methods). We found no reliable group differences in performance in either task (Volle 1s interval: M_High−symptom_= −6.578, SD_High−symptom_ = 12.902 vs. M_Low−symptom_ = −7.202, SD_Low−symptom_ = 13.354, *t*_(148)_ = 0.279, *p* = 0.78, d_z_ = 0.047; Volle 2s interval: M_High−symptom_ = −11.38, SD_High−symptom_ = 16.275 vs. M_Low−symptom_ = -12.086, SD_Low−symptom_ = 17.396, *t*_(148)_ = 0.247, *p* = 0.805, d_z_ = 0.042; Zauberman: M_High−symptom_= −0.324, SD_High−symptom_ = 1.77 vs. M_Low−symptom_= 1.172, SD_Low−symptom_ = 11.963, *t*_(142)_= -0.904, *p* = 0.368, d_z_ = 0.156) nor correlations with anorexia nervosa symptom severity (Volle 1s interval and EAT-26: *r*_(148)_ = 0.049, *p* = 0.549; Volle 2s interval and EAT-26: *r*_(148)_ = 0.023, *p* = 0.781; Zauberman and EAT-26: *r*_(142)_=-0.058, *p* = 0.49).

### Controlling for mood and anxiety

As expected, participants in the high anorexia nervosa symptom group also showed higher negative mood and anxiety symptoms as reflected by dispositional negativity scores compared to the low-symptom group (M_High−symptom_ = −0.411, SD_High−symptom_ = 0.962 vs. M_Low−symptom_ = −0.245, SD_Low−symptom_ = 0.944; *t*_(148)_ = 4.088, *p* < 0.001, d_z_= 0.69) (see Supplementary Fig. 2 & Supplementary Table 4). Accordingly, EAT-26 scores and dispositional negativity were positively correlated (*r*_(148)_ = 0.314, *p* < 0.001; Supplementary Fig. 3). In light of this group difference and potentially confounding factor, we next examined whether dispositional negativity was associated with intertemporal discounting and future orientation in our sample. Dispositional negativity was unrelated to intertemporal choice (*r*_(132)_ = 0.052, *p* = 0.551). Interestingly, dispositional negativity was negatively associated with future-orientation scores (*r*_(147)_= −0.290, *p* < 0.001), and these two factors accounted for independent variance in anorexia nervosa symptomatology, as follows. When entered in a simultaneous regression model, future orientation and dispositional negativity were both significant and independent predictors of EAT-26 scores (B_Future orientation_ = 3.825, SE = 1.026, *t* = 3.729, *p* < 0.001; B_Dispositional Negativity_ = 4.973, SE = 1.004, *t* = 4.954, *p* < 0.001) (Supplementary Fig. 4). Collectively, these analyses suggest that future-oriented cognition, and mood and anxiety symptomatology, while both linearly associated with severity of anorexia nervosa symptoms, independently explained anorexia nervosa symptomatology. Moreover, only future-oriented cognition, and not dispositional negativity, was associated with intertemporal choice in this sample.

### Controlling for gender, age, and income

Gender, age, and income themselves might influence intertemporal discounting behavior, anorexia nervosa symptomatology and future-oriented cognition. Therefore, we next examined whether gender, age and income differed as a function of each of these factors. First, we did not find that gender or income modulated delay discounting, future oriented cognition, or that they were differentially associated with anorexia nervosa symptomatology in our sample: Gender: log(*k*): *t*_(131)_ = −0.476, *p* = 0.635; future-orientation: *t*_(144)_ = 0.207, *p* = 0.837; EAT-26: *t*_(147)_ = −0.776, *p* = 0.439; Income: log(*k*): *F*_(6, 99)_ = 0.743, *p* = 0.616; future-orientation: *F*_(6, 109)_ = 1.324, *p* = 0.253; EAT-26: *F*_(6, 112)_ = 1.211, *p* = 0.306). Age was not associated with delay discounting (r_(133)_ = 0.09, *p* = 0.299). However, age was positively associated with future orientation (*r*_(146)_ = 0.241, *p* = 0.003) and with anorexia nervosa symptomatology (*r*_(149)_ = 0.222, *p* = 0.006); accordingly, individuals in the high-symptom group were significantly older than in the low-symptom group (*t*_(150)_ = 3.1372, *p* = 0.002).

Next, we examined whether anorexia nervosa symptomatology continued to predict intertemporal discounting after controlling for gender, age, and income. We found that anorexia nervosa symptomatology continued to predict reduced delay discounting (*F*_(1, 95)_ = 4.854, *p* = 0.03) and greater future-oriented cognition (*F*_(1, 105)_ = 9.919, *p* = 0.002) after entering gender, age, and income as covariates in an ANOVA model. Next, we examined whether future orientation continued to mediate the association between anorexia nervosa symptomatology and delay discounting behavior after controlling for gender, age, and income. To that end, we ran a mediation model with future-orientation factor scores as mediator and gender, age and income as covariates in the same model. We found that a future-oriented cognitive style continued to predict reduced intertemporal discounting, log(*k*) (ß = -0.319, SE = 0.153, *t* = −3.180, *p* = 0.002). A bootstrap analysis of the partial standardized indirect effect (ß = −0.164, SE = 0.086) provided evidence for a significant mediation [95% CI = (−0.354, −0.018)], whereby future orientation continued to mediate anorexia nervosa group differences in intertemporal discounting. Accordingly, after controlling for future orientation scores, gender, age, and income, the anorexia nervosa group difference in log(*k*) was reduced (total effect: ß =−0.467, SE = 0.324, *t* = −2.186, *p* = 0.031, partial effect: ß = −0.304, SE = 0.319, *t* = −1.440, *p* = 0.153). In sum, our findings were replicated after controlling for gender, age, and income.

### Further ascertaining the sensitivity of delay discounting to anorexia (vs. bulimia) nervosa symptoms

After excluding bulimia nervosa symptoms from EAT-26 final scores (see Methods), we replicated our results, as follows: higher anorexia nervosa symptomatology continued to predict lower intertemporal discounting (M_High−symptom_= −4.495, SD_High−symptom_ = 1.381 vs. M_Low−symptom_= −3.905, SD_Low−symptom_ = 1.520, *t*_(134)_= −2.182, *p* = 0.015, d_z_ = 0.4) and higher future-oriented cognition (M_High−symptom_= 0.314, SD_High−symptom_ = 1.005 vs. M_Low−symptom_= −0.127, SD_Low−symptom_ = 0.922, *t*_(147)_ = 2.674, *p* = 0.004, d_z_  = 0.464). Moreover, future-oriented cognition continued to mediate the association between anorexia nervosa symptoms and intertemporal discounting [ß = −0.105, SE = 0.063, 95% CI = (−0.253, −0.010)].

## Discussion

Eating disorders, including anorexia nervosa, are highly prevalent, challenging to treat, and associated with one of the highest mortality rates of all psychiatric conditions^75^. Yet, the neurocognitive mechanisms that drive the persistent overriding of food in the service of long-term goals in anorexia nervosa remain unclear. Here, we replicated and extended previous findings indicating reduced intertemporal discounting in patients with acute anorexia nervosa^13–17^ in a community sample of never diagnosed individuals. Moreover, our results suggested a neurocognitive mechanism that could underlie the propensity of individuals with anorexia to value delayed (vs. present-moment) rewards: temporal orientation. Specifically, we found that anorexia nervosa symptomatology was associated with a more future-oriented cognitive style. These results were unrelated to subjective time perception and held independently of mood and anxiety differences between high and low anorexia nervosa symptom groups. In summary, our study indicates that a future-oriented cognitive style is associated with anorexia nervosa symptomatology, which may contribute to reduced intertemporal discounting behavior in this population.

The propensity to prefer larger future rewards—as opposed to smaller-but-sooner rewards—is thought to depend on distinct yet mutually interactive systems: reward valuation, cognitive control, and prospective systems^2,3,23,64,76,77^. The prospective system is thought to interface with the valuation system with the potential to reduce myopic decision making, thereby promoting decisions that are consistent with increased cognitive control. Specifically, engaging in episodic future thinking is associated with reduced delay discounting and a shift away from hedonic choices in health domains, such as smoking and drinking behavior^43,44^ (reviewed in^2^). Put differently, engaging the prospective system, which relies on extended memory regions including the hippocampus, precuneus, and frontal pole, is thought to facilitate future-minded decision making^3,78,79^. Accordingly, in our study, future-oriented cognition correlated negatively with delay discounting across individuals. However, whether future-oriented cognition might contribute to a previously-reported imbalance of self-control and hedonic reward valuation systems in patients with anorexia nervosa^8,9^—and explain reduced delay discounting in this population^4^—had not previously been tested. Here, we show a robust difference in future-oriented cognition in individuals with higher anorexia symptomatology (compared to a low-symptom group). Further corroborating the potential relevance of this phenotype for eating disorders, we found that across individuals, anorexia symptoms (EAT-26 scores) correlated positively with future-oriented cognition. Moreover, future-oriented cognition mediated the association between anorexia nervosa symptomatology and reduced intertemporal discounting. Collectively, these results suggest that larger/delayed (vs. smaller/present-moment) rewards may be valued more by individuals with anorexia nervosa vulnerability in part due to the propensity of those individuals to spontaneously consider the future during their everyday life decisions. Overall, these insights align with prior accounts suggesting an imbalance of reward valuation versus control systems in patients with anorexia nervosa^8,9^—and offer a unified cognitive mechanism that may underlie precisely such an imbalance.

Current and previous findings suggesting that future rewards hold higher value among individuals with anorexia nervosa symptomatology contrast markedly with what is typically seen in other psychiatric illnesses characterized by low levels of self-control, as indexed by higher intertemporal discounting—such as in substance abuse disorders—illustrating that intertemporal discounting may be a useful marker of (mal)adaptive cognition at both ends of the spectrum^2,4,33^. An increased ability to delay rewards may contribute to disordered behaviors in patients with anorexia nervosa, such as persistent dieting and food restriction, via the overvaluation of future goals. It is possible that individuals who habitually engage in future thinking view future rewards as more concrete, which has been shown to increase their relative value, and be associated with less discounting^41,45^. As mentioned above, the ability to vividly imagine future consequences and action outcomes has been suggested to be a proximal mechanism by which prospective thinking promotes future-oriented decision making and facilitates cognitive control over actions in the present^5,80^. Therefore, future work should examine whether imagery of future outcomes may be more vivid and/or concrete in individuals with anorexia nervosa. Relatedly, results from a recent study suggest that increased engagement of extended memory neural systems during the viewing of thinness-related pictures predicts the persistence of eating pathology^81^. Thus, future research could also examine whether prospective memory systems are more functionally connected with valuation and cognitive control systems in individuals with anorexia nervosa compared to healthy controls, which may underlie increased future-oriented thinking and decision making in this population.

Over time, behaviors that begin as future goal-oriented actions may transition into habitual, automatic behaviors that are relatively insensitive to action outcome, putatively via reinforcement learning mechanisms^82^. Individuals with anorexia nervosa show stronger habit-like, automatic behaviors as indexed by self-reported questionnaires (relative to healthy controls), the strength of which correlates positively with the severity and duration of the disease^83^. Consistently, in a novel virtual reality task designed to measure behavioral trajectories of approach and avoidance, patients with the restrictive subtype of anorexia nervosa show stronger habitual food avoidance relative to healthy controls (in addition to heightened inhibitory control)^84^. Therefore, it is possible that future-oriented cognition may become particularly problematic if coupled with the subsequent formation of persistent, habitual food avoidance behavior in individuals with anorexia nervosa vulnerability^85,86^. Prospective longitudinal studies will be required to examine whether and how future-oriented cognition and habitual food avoidance processes inter-relate or whether they may represent distinct illness factors and risk trajectories for different individuals.

The following limitations from the current study warrant further investigation. First, the data for our study were collected online (beginning during the COVID-19 pandemic). Given the inherent challenges associated with providing adequate participant monitoring and timely psychological assistance in an online data collection environment, we removed what we considered sensitive questions from our study for ethical reasons—including questions about participants’ BMI. Therefore, although our sample may be considered ‘never-diagnosed’ in the sense that participants self-reported not having been previously diagnosed with an eating disorder, it is possible (and likely) that the anorexia nervosa high-symptom group included individuals who met clinical diagnostic criteria for anorexia nervosa, which we would have been better equipped to determine had their BMI data been collected or a formal clinical diagnostic interview conducted. Thus, the present study leaves unclear the extent to which our sampling strategy captured primarily vulnerability for anorexia nervosa versus whether our sample comprised a mixture of both at-risk (subclinical) and clinical cases. Relatedly, in the absence of BMI information, our study was unable to fully differentiate between anorexia nervosa and bulimia nervosa symptomatology, which are known to be differentially liked to intertemporal discounting, with acute anorexia nervosa predicting reduced, and bulimia nervosa predicting increased intertemporal discounting behavior compared to healthy controls^4,33^ (of note, analyses excluding bulimia-nervosa related items from EAT-26 scores prior to symptom-based group assignment replicated our original results). In summary, future work is needed to determine whether the present findings replicate in a strictly at-risk sample of individuals presenting anorexia nervosa symptoms.

It is important to note that the present study had a cross-sectional design—therefore, we are unable to infer causality in the association between future orientation, intertemporal discounting, and anorexia nervosa symptomatology. On the one hand, the finding that individuals with higher anorexia symptomatology show reduced delay discounting compared to a low-symptom group extends prior findings reported in patients with acute anorexia nervosa^13–17^ to a not-previously diagnosed sample—suggesting that altered future-oriented cognition and decision making could be a vulnerability factor for anorexia nervosa (for meta-analyses, see^2,4^). However, a prior longitudinal study did not find altered delayed discounting in individuals with anorexia nervosa following treatment and remission^15^ (for related null findings in patients with remitted anorexia nervosa symptoms, see^17,34,35^). Therefore, future work tracking individuals at risk for anorexia prospectively and longitudinally—with repeated measurements of future-oriented cognition and intertemporal discounting behavior—will be required to ascertain causality and determine whether alterations in intertemporal thinking and decision making are merely correlates of anorexia nervosa symptomatology, or whether they may be a vulnerability factor that could be proactively targeted prior to anorexia nervosa onset^87^. If so, interventions that alter construal level (from more concrete to more abstract) have been shown to modulate the value ascribed to proximal versus distal rewards^88^, and could be promising future avenues for treatment.

In light of prior work suggesting a role for subjective time perception in delay discounting and impulsive decision making^38,39,49–52^, we included assessments of time perception in our study, which were examined in relation to anorexia nervosa symptomatology in pre-registered exploratory analyses. Contrary to our hypotheses, we did not find that time was experienced faster (and/or more accurately) in individuals with higher anorexia nervosa symptoms. This contrasts with a previous report in patients with acute anorexia, which found accelerated time estimates in clinically diagnosed individuals compared to controls^53^. Collectively, these findings suggest that while temporal factors do play an underappreciated and seldom-studied role in the pathophysiology of anorexia nervosa, it is primarily the valuation of rewards and consequences over time—into the future—that is associated with anorexia nervosa symptomatology, rather than a change in one’s internal clock^39^.

In closing, our results demonstrate that individuals with higher anorexia nervosa symptomatology are *willing to wait*, showing greater future-oriented cognition and decision making compared to a low-symptom group. Together, these findings pave the way for longitudinal studies with at-risk samples to elucidate the import of future-oriented cognition and delay discounting as potential neurocognitive markers of anorexia nervosa, and determine the role of prospective neural network systems for anorexia nervosa vulnerability.

## Electronic supplementary material

Below is the link to the electronic supplementary material.

Supplementary Material 1

## Data Availability

This study was preregistered on OSF (https://osf.io/h46x2*).* Any discrepancies between the preregistered plan and reported analyses are described in Supplementary Materials. Study materials, including the data, scripts used for running the experiment, and data analysis scripts, can be found on OSF: https://osf.io/3y7dq/.
