# Author Correction: Willing to wait: Anorexia nervosa symptomatology is associated with higher future orientation and reduced intertemporal discounting

**DOI:** 10.1038/s41598-025-16462-y

**Published:** 2025-08-25

**Authors:** Isabel Schuman, Jingyi Wang, Ian C. Ballard, Regina C. Lapate

**Affiliations:** 1 Department of Psychological & Brain Sciences, University of California, Santa Barbara, Santa Barbara, USA; 2 Department of Psychology, University of California, Riverside, Riverside, USA

Correction to: *Scientific Reports* 10.1038/s41598-024-80597-7, published online 06 February 2025

The original version of this Article contained an error in Figure 2, panel B, which was inadvertently replaced with Supplementary Figure 2.

The incorrect Fig. 2 and its legend appear below.

**Fig. 2:**
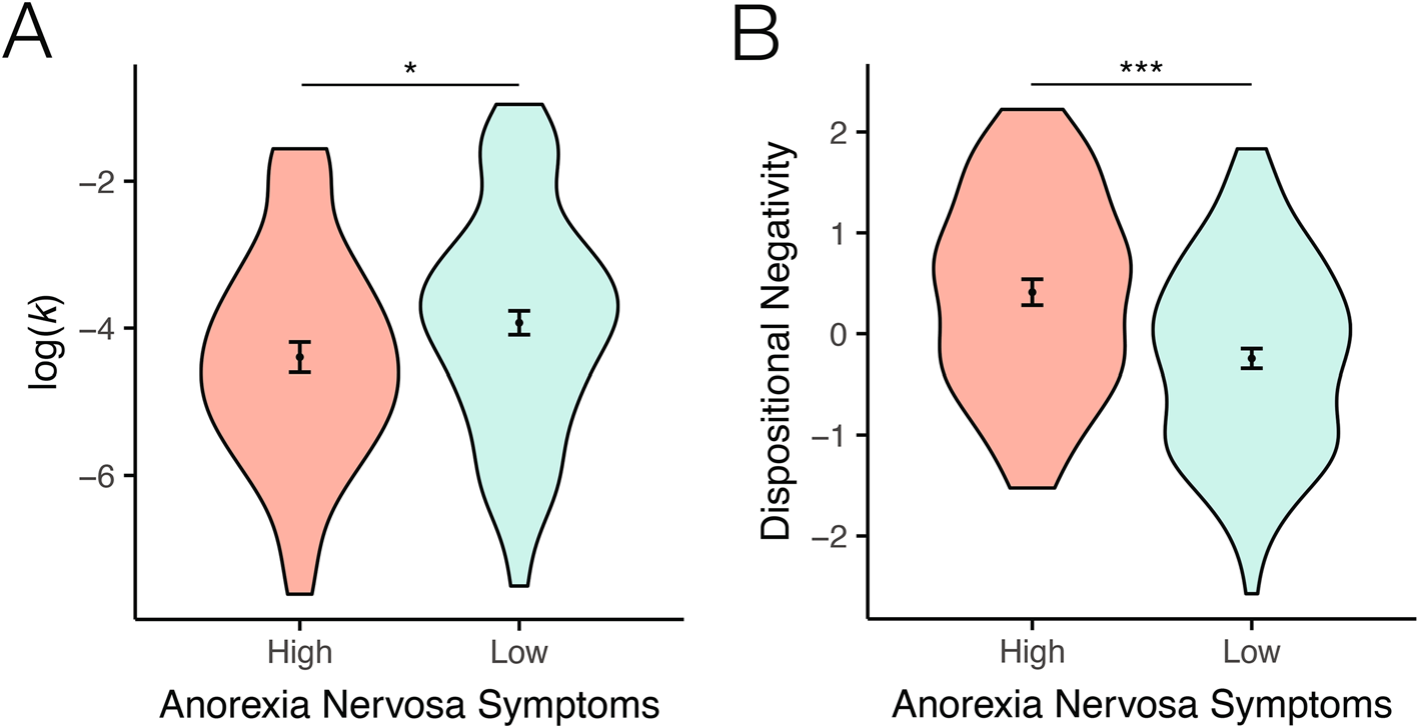
Intertemporal decision making (**A**) and temporal orientation (**B**) as a function of anorexia nervosa symptoms. (**A**) Individuals with higher anorexia nervosa symptoms show reduced delay discounting compared to a low-symptom group. (**B**) Anorexia nervosa symptomatology is associated with a future-oriented cognitive style. *p < 0.05, ***p < 0.005.

The original Article has been corrected.

