# Supplementary material for: Willing to wait: Anorexia nervosa symptomatology is associated with higher future orientation and reduced intertemporal discounting
Source: Sci Rep. 2025 Feb 6;15:4508. doi: 10.1038/s41598-024-80597-7 (PMC11802882; doi:10.1038/s41598-024-80597-7)
Supplement: Supplementary file 1 — Supplementary Material 1 [file 41598_2024_80597_MOESM1_ESM.pdf]

Supplementary Materials

Additional analyses beyond the pre-registration

Analyses that were not part of the preregistered plan (https://osf.io/h46x2) are listed below.

Control analysis with dispositional negativity. Individuals with anorexia have a higher propensity to experience negative affect and anxiety[1,2]. Therefore, we administered mood and anxiety questionnaires (and derived a Dispositional Negativity factor score, as detailed in the *Methods*) to disentangle the specific contributions of future-orientation cognition versus mood and anxiety symptomatology to anorexia nervosa symptomatology.

Mediation analysis between anorexia nervosa group, future orientation, and intertemporal discounting. The results from our pre-registered analyses indicated that individuals in the high-symptom group devalued future rewards less and were more future oriented than individuals in the low-symptom group. Given that episodic future thinking has been postulated to be an important cognitive factor that underlies intertemporal behavioral choices[3], we conducted a mediation analysis to test the hypothesized directional link between anorexia nervosa symptomatology, future orientation, and intertemporal discounting.

Testing of the sensitivity of delay discounting metrics with anorexia nervosa symptomatology. We used pre-registered EAT-26 questionnaire scores as a metric to index anorexia nervosa symptomatology in our current sample, which showed differences in delay discounting behavior and future-oriented cognition between anorexia (high *vs* low) symptom groups. However, recent studies indicate that individuals with Anorexia versus Bulimia often show opposite patterns of delay-discounting behaviors[4,5], such that Anorexia is linked to *reduced* delay discounting, and Bulimia to *increased* delay discounting, compared to healthy controls. Therefore, although the EAT-26 questionnaire was not originally designed to discriminate between the two populations[6], we performed an additional control analysis that excluded EAT-26 items that explicitly indexed bulimia symptomatology (e.g., binge-eating and purging behavior) from the final EAT-26 score—prior to symptom-based group assignment—to ascertain the specificity of the associations we uncovered between anorexia nervosa symptomatology as assessed by the EAT-26, delay discounting, and future-oriented cognition to anorexia nervosa.

Supplementary Methods

Data quality exclusion criteria

Delay discounting task. Following our pre-registered plan to maximize data quality and verify task compliance in this online study, data quality checks (i.e., catch trials) were included in the delay discounting task, and low-quality data (due to insufficient data and/or unreliable model fits) were excluded from analyses, as follows. We administered randomly placed catch trials that asked participants to choose between a "smaller later" reward and a "larger sooner" reward (e.g., \$5 in two weeks *vs.* \$10 today; see *Supplementary Table 2*). We excluded participants from analysis of the delay discounting task if they failed a catch trial (e.g., "smaller later" reward), and/or if their model fitting raised a warning due to random responding behavior detected by the hierarchical Bayesian inference model (cf.[7]). Eight (out of 152) participants failed a catch trial, and 8 (out of the remaining 144) participants' hierarchical hyperbolic model fits raised a random-responding warning. Thus, a total of n = 16/152 participants were excluded due to poor-quality data in the delay discounting task, rendering the final sample n = 136 (Age *M* = 27.18, *SD* = 9.46; n = 97 females, 36 males, 3 non-binary; n = 49 of those individuals fell in the high-symptom group (Age *M* = 30.8, *SD* = 10.16; n = 37 female, 12 male) and n = 87 in the low-symptom group (Age *M* = 25.12, *SD* = 8.43; n = 60 female, 27 male)).

Future Orientation questionnaire. Following our pre-registered plan, participants' questionnaire data were excluded if they failed to respond to at least 75% of a questionnaire's items and/or if their score exceeded 3 SD from the mean. Three participants (out of n = 152 eligible participants) were excluded from analysis following this criterion; one participant due to insufficient responses in the Preoccupation with Future Events Questionnaire, one participant due to insufficient responses in the Consideration of Future Consequences, and one participant due to outlying values in the future-orientation factor scores, rendering the final sample n = 149 for future orientation related analyses (Age *M* = 27.22, *SD* = 9.66; n = 107 females, 39 males, 3 non-binary; n = 55 of those individuals fell in the high-symptom group (Age *M* = 30.2, *SD* = 10.04; n = 42 female, 13 male) and n = 94 in the low-symptom group (Age *M* = 25.46, *SD* = 9.03; n = 65 female, 29 male), *Supplementary Table 1*).

The Ebert-Prelec model

The Ebert-Prelec model assumes that subjective values are calculated according to:

$$SV = e^{-(at)^b}$$

Where a reflects impatience (similarly to k in the hyperbolic model) and b reflects time-sensitivity. Subjective values influenced choices according to the *softmax* decision function:

$$p(Choose\ LL) = \frac{e^{mSV_{LL}}}{e^{mSV_{LL}} + e^{mSV_{SS}}}$$

Where m is the inverse temperature controlling the sensitivity of choices to subjective value and SV_{LL} and SV_{SS} are the subjective values of the larger-later and smaller-sooner options, respectively. For prior probabilities of parameters, we used a uniform distribution for a and b and a 10*Beta(2,6) prior on m. We maximized the posterior probability of the data using conjugate gradient descent implemented in Scipy 1.7.1.

The Ebert-Prelec model accounted for 86.5% of subject choices. Because of this high accuracy, none of the subjects met our preregistered exclusion criterion of model performance that was indistinguishable from chance. However, examination of the parameter distributions indicated that there were outlier model fits at the extremes of the parameter space. Because these extreme parameter values are likely due to noise in the model fitting and not our psychological constructs of interest, we implemented an additional exclusion criterion by excluding subjects with a or b parameters greater than 3 SD outside of the mean. This excluded n = 16 subjects and resulted in a final sample of n = 136 participants.

Final sample counts

As detailed in the *Methods*, our pre-registered exclusion criteria included poor quality and/or outlier data, which were removed on a per-metric basis. The final sample counts and demographics for each analysis are listed in the *Supplementary Table 1*.

Supplementary Results

The Ebert-Prelec model

We examined whether there was a difference in impatience or time sensitivity between high-symptom and low-symptom groups. We found that the low-symptom group was more impatient than the high-symptom group, as measured by parameter *a* in the Ebert-Prelect model ($M_{High\text{-}symptom} = 0.013$, $SD_{High\text{-}symptom} = 0.02$ vs. $M_{Low\text{-}symptom} = 0.02$, $SD_{Low\text{-}symptom} = 0.027$, $t_{(134)} = -1.688$, $p = 0.047$, $d_z = 0.303$), replicating the results from the hyperbolic model. However the two groups did not show significant differences in time sensitivity, as measured by model parameter *b* ($M_{High\text{-}symptom} = 0.715$, $SD_{High\text{-}symptom} = 0.27$ vs. $M_{Low\text{-}symptom} = 0.791$, $SD_{Low\text{-}symptom} = 0.308$, $t_{(134)} = -1.438$, $p = 0.076$, $d_z = 0.258$). In addition, because of the non-normality of the *a* and *b* distributions, we used Mann-Whitney U tests to compare groups. Consistently, we found that the low-symptom group was more impatient than the high-symptom group ($U = -1.83$, $p = 0.033$). However, we found no difference between groups in time sensitivity ($p > 0.1$).

Following our pre-registered correlation analysis, we examined whether the Ebert-Prelec parameters indexing impatience (*a*) or time sensitivity (*b*) were associated with delay discounting and/or future orientation; we did not find significant associations (*a* and EAT-26 score: $r_{(134)} = -0.082$, $p = 0.171$; *b* and EAT-26 score: $r_{(134)} = -0.124$, $p = 0.151$; *a* and future-orientation: $r_{(133)} = -0.139$, $p = 0.054$; *b* and future-orientation: $r_{(133)} = -0.031$, $p = 0.719$).

Associations between EAT questionnaire scores, future orientation, and intertemporal discounting

Interestingly, even though anorexia nervosa (high vs. low) symptom groups differed in intertemporal discounting—with lower delay discounting observed in the high-symptom group (see main text)—the linear correlation between EAT questionnaire scores and log(*k*) was not significant (log(*k*) and EAT: $r_{(134)} = -0.046$, $p = 0.298$) suggesting potential non-linearities in the association between anorexia nervosa symptomatology and intertemporal choice behavior. In other words, it is possible that delay discounting differences are only evident and/or meaningful for anorexia nervosa once individuals reach a clinically-relevant threshold (such as used to define high symptom groups in this and other studies). In contrast, EAT scores were linearly (and positively) associated with future-oriented cognition ($r_{(147)} = 0.18$, $p = 0.014$) (*Supplementary Figure 1*).

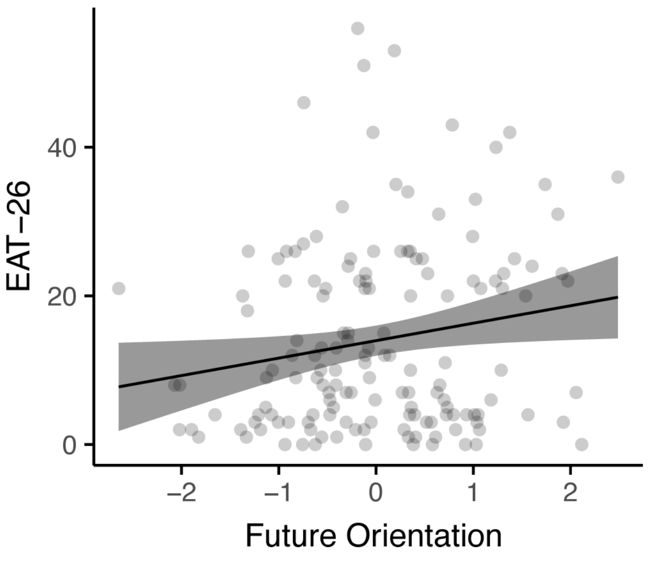

Supplementary Figure 1. The association between EAT-26 scores and Future Orientation is shown.

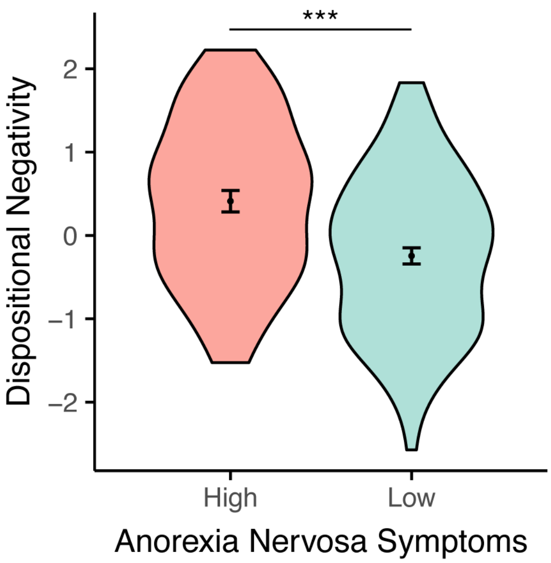

Supplementary Figure 2. Individuals with higher anorexia symptomatology higher mood and anxiety symptoms (Dispositional Negativity factor score). ***$p < 0.005$

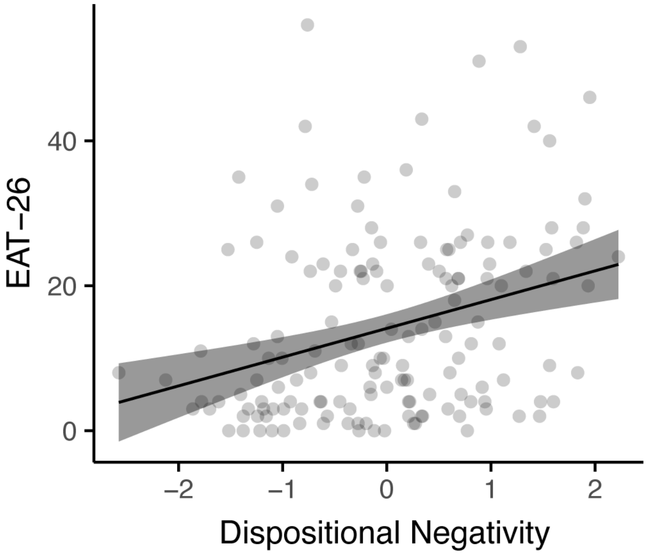

Supplementary Figure 3. The association between EAT-26 scores and Dispositional Negativity is shown.

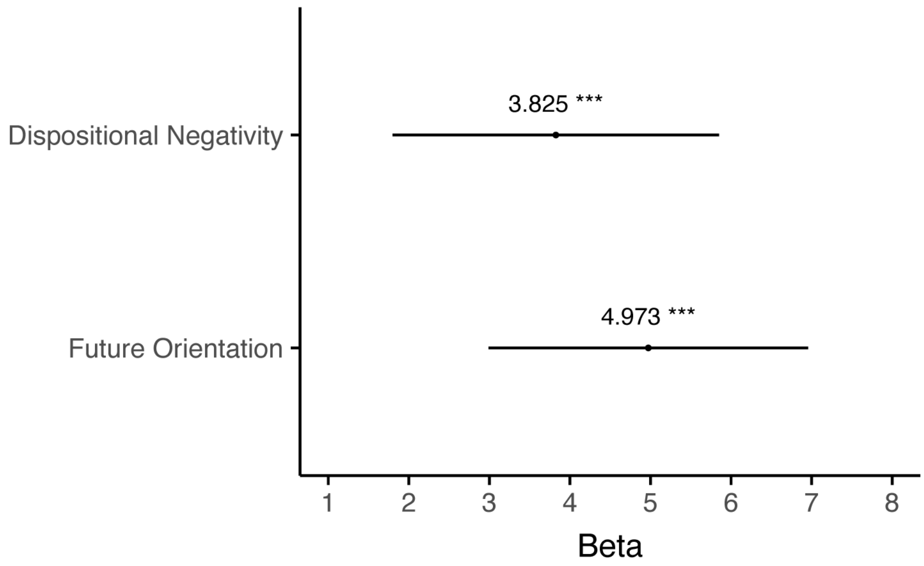

Supplementary Figure 4. Dispositional negativity and future orientation independently predict anorexia nervosa symptomatology (EAT-26 scores). Fixed-effect estimates are plotted. *** $p < 0.001$.

***Supplementary Table 1*. Demographic information for individual analyses**

Analysis	Sample n	Age range	Age Mean	Age SD	Female	Male	Non-binary	High-symptom	Low-symptom
log(*k*) by AN	136	18-51	27.18	9.46	97	36	3	49	87
log(*k*) by FO	134	18-51	27.01	9.41	96	35	3	47	87
FO by AN	149	18-51	27.22	9.66	107	39	3	55	94
Volle by AN	150	18-51	27.25	9.68	107	40	3	55	95
Zauberman by AN	144	18-51	27.50	9.80	101	40	3	53	91
DN by AN	150	18-51	27.27	9.64	108	39	3	56	94
DN by FO	149	18-51	27.22	9.66	107	39	3	55	94
FO by log(*k*) by AN	134	18-51	27.01	9.41	96	35	3	47	87
FO by DN by AN	149	18-51	27.22	9.66	107	39	3	55	94

log(*k*): temporal discounting rate. AN (Anorexia Nervosa): EAT-26 score. FO: future-orientation factor score. Volle: errors for 1s and 2s interval conditions in the Volle task. Zauberman: errors in the Zauberman task. DN: dispositional negativity scores. "Metric1" by "Metric2": two sample t-test and/or Pearson's correlation coefficient for "Metric1" and "Metric2". AN by FO by log(*k*): mediation test for future-orientation factor scores (mediating the association between EAT-26 scores and temporal discounting). AN by FO by DN: simultaneous regression model with dispositional negativity scores and future-orientation scores predicting EAT-26 scores.

Supplementary Table 2. **Catch trials included in the delay discounting task**

SS	LL	STIME	LTIME	Platform
10	5	Today	Two weeks	SONA
10	5	Today	Two weeks	SONA
10	5	Today	Two weeks	Prolific
10	5	Today	Two weeks	Prolific
20	10	Today	Two weeks	Prolific
20	10	Today	Two weeks	Prolific
30	5	Today	Two weeks	Prolific
30	5	Today	Two weeks	Prolific
35	10	Today	Six Months	Prolific
35	10	Today	Six Months	Prolific
54	15	Today	Six Months	Prolific
54	15	Today	Six Months	Prolific

***Supplementary Table 3*. Intertemporal choices in the delay discounting task**

SS	LL	STIME	LTIME	Relative DIFF
55	56	Today	One week	0.02
58	60	Today	One week	0.03
62	66	Today	One week	0.06
56	60	Today	One week	0.07
44	48	Today	One week	0.09
69	79	Today	One week	0.14
34	41	Today	One week	0.21
29	36	Today	One week	0.24
72	97	Today	One week	0.35
57	86	Today	One week	0.51
36	63	Today	One week	0.75
58	116	Today	One week	1.00
38	39	Today	Two weeks	0.03
36	37	Today	Two weeks	0.03
80	85	Today	Two weeks	0.06
42	45	Today	Two weeks	0.07
41	45	Today	Two weeks	0.10
43	49	Today	Two weeks	0.14
36	43	Today	Two weeks	0.19
59	74	Today	Two weeks	0.25
42	57	Today	Two weeks	0.36
20	30	Today	Two weeks	0.50
28	49	Today	Two weeks	0.75
62	124	Today	Two weeks	1.00
53	54	Today	One month	0.02
32	33	Today	One month	0.03
18	19	Today	One month	0.06
73	79	Today	One month	0.08
72	79	Today	One month	0.10
67	77	Today	One month	0.15
44	53	Today	One month	0.20
47	59	Today	One month	0.26
46	62	Today	One month	0.35
40	60	Today	One month	0.50
53	93	Today	One month	0.75
79	158	Today	One month	1.00
36	37	Today	Two months	0.03
29	30	Today	Two months	0.03

40	42	Today	Two months	0.05
72	78	Today	Two months	0.08
84	92	Today	Two months	0.10
68	78	Today	Two months	0.15
72	86	Today	Two months	0.19
71	89	Today	Two months	0.25
17	23	Today	Two months	0.35
83	124	Today	Two months	0.49
33	58	Today	Two months	0.76
20	40	Today	Two months	1.00
42	43	Today	Three months	0.02
16	17	Today	Three months	0.06
65	69	Today	Three months	0.06
69	75	Today	Three months	0.09
58	64	Today	Three months	0.10
49	56	Today	Three months	0.14
28	34	Today	Three months	0.21
85	106	Today	Three months	0.25
67	90	Today	Three months	0.34
79	118	Today	Three months	0.49
84	147	Today	Three months	0.75
38	76	Today	Three months	1.00
39	40	Today	Six months	0.03
15	16	Today	Six months	0.07
43	46	Today	Six months	0.07
83	90	Today	Six months	0.08
22	24	Today	Six months	0.09
34	39	Today	Six months	0.15
50	60	Today	Six months	0.20
70	88	Today	Six months	0.26
41	55	Today	Six months	0.34
65	98	Today	Six months	0.51
74	130	Today	Six months	0.76
46	92	Today	Six months	1.00
81	83	Two weeks	One month	0.02
76	79	Two weeks	One month	0.04
28	30	Two weeks	One month	0.07
81	87	Two weeks	One month	0.07
34	37	Two weeks	One month	0.09
51	59	Two weeks	One month	0.16

53	64	Two weeks	One month	0.21
83	104	Two weeks	One month	0.25
50	68	Two weeks	One month	0.36
29	44	Two weeks	One month	0.52
16	28	Two weeks	One month	0.75
59	118	Two weeks	One month	1.00
25	26	Two weeks	Two months	0.04
39	41	Two weeks	Two months	0.05
49	52	Two weeks	Two months	0.06
36	39	Two weeks	Two months	0.08
51	56	Two weeks	Two months	0.10
77	89	Two weeks	Two months	0.16
24	29	Two weeks	Two months	0.21
69	86	Two weeks	Two months	0.25
33	45	Two weeks	Two months	0.36
75	112	Two weeks	Two months	0.49
80	140	Two weeks	Two months	0.75
39	78	Two weeks	Two months	1.00
34	35	Two weeks	Three months	0.03
83	86	Two weeks	Three months	0.04
59	63	Two weeks	Three months	0.07
35	38	Two weeks	Three months	0.09
63	69	Two weeks	Three months	0.10
48	55	Two weeks	Three months	0.15
31	37	Two weeks	Three months	0.19
23	29	Two weeks	Three months	0.26
23	31	Two weeks	Three months	0.35
81	122	Two weeks	Three months	0.51
45	79	Two weeks	Three months	0.76
85	170	Two weeks	Three months	1.00
26	27	One month	Two months	0.04
31	32	One month	Two months	0.03
24	25	One month	Two months	0.04
48	52	One month	Two months	0.08
68	75	One month	Two months	0.10
65	75	One month	Two months	0.15
79	95	One month	Two months	0.20
26	32	One month	Two months	0.23
52	70	One month	Two months	0.35
33	50	One month	Two months	0.52

65	114	One month	Two months	0.75
34	68	One month	Two months	1.00
63	64	One month	Three months	0.02
54	56	One month	Three months	0.04
85	90	One month	Three months	0.06
25	27	One month	Three months	0.08
18	20	One month	Three months	0.11
31	36	One month	Three months	0.16
45	54	One month	Three months	0.20
27	34	One month	Three months	0.26
45	61	One month	Three months	0.36
81	122	One month	Three months	0.51
80	140	One month	Three months	0.75
76	152	One month	Three months	1.00
47	48	One month	Six months	0.02
52	54	One month	Six months	0.04
22	23	One month	Six months	0.05
15	16	One month	Six months	0.07
81	89	One month	Six months	0.10
42	48	One month	Six months	0.14
27	32	One month	Six months	0.19
34	42	One month	Six months	0.24
64	86	One month	Six months	0.34
84	126	One month	Six months	0.50
34	60	One month	Six months	0.76
74	148	One month	Six months	1.00

SS: smaller-sooner rewards, LL: larger-later rewards, STIME: time until receipt of the smaller-sooner rewards, LTIME: time until receipt of the larger-later rewards, Relative DIFF: The relative difference in reward magnitudes between smaller-sooner and larger-later rewards.

***Supplementary Table 4.* Descriptive statistics [*M (SD)*] of key demographic and experimental variables examined in the current study for high and low anorexia nervosa symptom groups**

	**Anorexia Nervosa Symptomatology**	
**Metric**	**High**	**Low**
Age	30.49 (10.01)	25.41 (8.99)
EAT-26	28.179 (8.894)	5.745 (4.441)
log(*k*)	-4.394 (1.434)	-3.928 (1.514)
Delay discounting model (Alpha)	3.792	3.144
Delay discounting model (Epsilon)	0.071	0.06
Future orientation (factor score)	0.292 (1.007)	-0.138 (0.916)
Zauberman task score	-0.324 (1.77)	1.172 (11.963)
Volle task score (1s)	-6.578 (12.902)	-7.202 (13.354)
Volle task score (2s)	-11.38 (16.275)	-12.086 (17.396)
Dispositional negativity (factor score)	0.411 (0.962)	-0.245 (0.944)

***Supplementary Table 5*. Summary of validity and reliability metrics for questionnaires used in this study**

**Questionnaire**	**Validity**	**Reliability & Internal Consistency**	**Internal Consistency for Current Sample (Cronbach's alpha)**
EAT-26	83.6% correct classification rate for patients with anorexia nervosa[8]	Cronbach's alpha: 0.82-0.9[8]	0.898
ZPTI (future)	Correlation with CFC (r = 0.52, $p < 0.001$)[9]	Cronbach's alpha: 0.77[9]	0.799
CFC	Correlation with Ray and Najman's deferment of gratification scale (r = 0.47, $p < 0.001$)<br><br>Correlation with internal-external locus of control scale (r = 0.25, $p < 0.01$)<br><br>Correlation with ZTPI: future orientation subscale (r = 0.43, $p < 0.01$)[10]	Cronbach's alpha: 0.8-0.86[10]	0.766
BDI	60%-72% correct classification rate for clinical depression[11–13]	Test-retest reliability r = 0.93, $p < 0.001$[14]<br><br>Cronbach's alpha: 0.92 for psychiatric outpatients and 0.93 for college students[15]	0.921

STAI-T	Kaiser-Meyer-Olkin score (0.824) indicating good construct validity[16]	Test-retest coefficients for STAI-T ranging from 0.69 to 0.89[17]<br><br>Cronbach's alpha: 0.86 - 0.95[17]	0.935
ATQ (negative affect subscale)	ATQ-NA subscale Correlation with Neuroticism (Big Five) ($r = 0.74$)[18]	Test-retest coefficients: fear: 0.75; sadness: 0.73; discomfort: 0.6; frustration: 0.75[19]<br><br>Cronbach's alpha: fear: 0.71; sadness: 0.64; discomfort: 0.61; frustration: 0.69[19]<br><br>Cronbach's alpha: 0.81[18]	0.589

***Supplementary Table 6*. Future orientation metrics: Comparison between low and high anorexia nervosa symptom groups**

	**Anorexia Nervosa Symptomatology**		***t*-value**	***p*-value**
**Metric**	**High**	**Low**		
ZTPI	**3.81 (0.64)***	**3.547 (0.593)**	**2.558**	**0.006**
CFC	18.714 (3.581)~	17.851 (3.716)	1.395	0.083
PFE	0.362 (0.217)~	0.31 (0.19)	1.549	0.062
Future orientation (ZTPI & CFC factor score)	**0.267 (1.004)***	**0.122 (0.923)**	**2.402**	**0.009**

Bold font and asterisks indicate significant differences (t-test $p < 0.05$) between high and low anorexia nervosa symptomatology groups. The "~" indicates numerically trending differences (t-test $p < 0.1$).

***Supplementary Table 7*. Correlation between delay discounting scores [log(*k*)] and individual future orientation metrics**

**Metric**	**Pearson's *r***	**Pearson's *p***
ZTPI	**-0.235 ***	**0.006**
CFC	**-0.218 ***	**0.011**
PFE	0.021	0.81
Future orientation (ZTPI & CFC factor score)	**-0.287 ***	**0.001**

Bold font and asterisks indicate statistically significant Pearson correlation coefficients ($p < 0.05$)

References

1. Harney, M. B., Fitzsimmons-Craft, E. E., Maldonado, C. R. & Bardone-Cone, A. M. Negative affective experiences in relation to stages of eating disorder recovery. *Eat. Behav.* **15**, 24–30 (2014).
2. Garcia, D., Granjard, A., Lundblad, S. & Archer, T. A dark past, a restrained present, and an apocalyptic future: time perspective, personality, and life satisfaction among anorexia nervosa patients. *PeerJ* **5**, e3801 (2017).
3. Lempert, K. M. & Phelps, E. A. The Malleability of Intertemporal Choice. *Trends Cogn. Sci.* **20**, 64–74 (2016).
4. Amlung, M. *et al.* Delay Discounting as a Transdiagnostic Process in Psychiatric Disorders: A Meta-analysis. *JAMA Psychiatry* **76**, 1176–1186 (2019).
5. Lempert, K. M., Steinglass, J. E., Pinto, A., Kable, J. W. & Simpson, H. B. Can delay discounting deliver on the promise of RDoC? *Psychol. Med.* **49**, 190–199 (2019).
6. Mintz, L. B. & O'Halloran, M. S. The Eating Attitudes Test: validation with DSM-IV eating disorder criteria. *J. Pers. Assess.* **74**, 489–503 (2000).
7. Vincent, B. T. Hierarchical Bayesian estimation and hypothesis testing for delay discounting tasks. *Behav. Res. Methods* **48**, 1608–1620 (2016).
8. Garner, D. M., Olmsted, M. P., Bohr, Y. & Garfinkel, P. E. The eating attitudes test: psychometric features and clinical correlates. *Psychol. Med.* **12**, 871–878 (1982).
9. Zimbardo, P. G. & Boyd, J. N. Putting time in perspective: A valid, reliable individual-differences metric. *J. Pers. Soc. Psychol.* **77**, 1271–1288 (1999).
10. Strathman, A., Gleicher, F., Boninger, D. S. & Edwards, C. S. The consideration of future consequences: Weighing immediate and distant outcomes of behavior. *J. Pers. Soc. Psychol.* **66**, 742–752 (1994).
11. Beck, A. T., Ward, C. H., Mendelson, M., Mock, J. & Erbaugh, J. An inventory for measuring depression. *Arch. Gen. Psychiatry* **4**, 561–571 (1961).
12. Jackson-Koku, G. Beck Depression Inventory. *Occup. Med.* **66**, 174–175 (2016).
13. Beck, A. T. & Clark, D. A. Anxiety and depression: An information processing perspective. *Anxiety Res.* **1**, 23–36 (1988).

14. Beck, A. T., Steer, R. A., Ball, R. & Ranieri, W. Comparison of Beck Depression Inventories -IA and -II in psychiatric outpatients. *J. Pers. Assess.* **67**, 588–597 (1996).
15. Beck, A. T., Brown, G., Steer, R. A., Eidelson, J. I. & Riskind, J. H. Differentiating anxiety and depression: A test of the cognitive content-specificity hypothesis. *J. Abnorm. Psychol.* **96**, 179–183 (1987).
16. Vitasari, P., Wahab, M. N. A., Herawan, T., Othman, A. & Sinnadurai, S. K. Re-test of State Trait Anxiety Inventory (STAI) among Engineering Students in Malaysia: Reliability and Validity tests. *Procedia - Social and Behavioral Sciences* **15**, 3843–3848 (2011).
17. Spielberger, C. D., Gorsuch, R. L., Lushene, R., Vagg, P. R. & & Jacobs, G. A. Manual for the state-trait anxiety inventory. *Consulting Psychologists Press* (1983).
18. Evans, D. E. & Rothbart, M. K. Developing a model for adult temperament. *J. Res. Pers.* **41**, 868–888 (2007).
19. Derryberry, D. & Rothbart, M. K. Arousal, affect, and attention as components of temperament. *J. Pers. Soc. Psychol.* **55**, 958–966 (1988).
